# Excretion Routes of Okadaic Acid and Dinophysistoxin-2 from Mussels (*Mytilus galloprovincialis*) and Cockles (*Cerastoderma edule*)

**DOI:** 10.3390/toxins17030128

**Published:** 2025-03-09

**Authors:** Juan Blanco, Noelia Estévez-Calvar, Helena Martín

**Affiliations:** Centro de Investigacións Mariñas, Xunta de Galicia, Pedras de Corón s/n, 36620 Vilanova de Arousa, Spain; noelia.estevez.calvar@xunta.gal (N.E.-C.); helena.martin.sanchez@xunta.gal (H.M.)

**Keywords:** biotoxins, bivalves, depuration, DSP, okadaic acid, dinophysistoxin-2, DTX2, excretion, esters

## Abstract

The knowledge of the routes of excretion of the toxins accumulated by molluscs is a key step in designing methods that accelerate depuration. In this work, the excretion route, in mussels and cockles, of the main diarrhetic shellfish poisoning (DSP) toxins in Europe (okadaic acid and dinophysistoxin-2) after natural intoxication were studied. During depuration, the amounts of free toxins and their derivatives were quantified in bivalves, faeces, and water. Most toxins (>98%) were excreted through faeces as acyl derivatives (most likely 7-*O-*acyl esters), independent of the ratio between these derivatives and free toxins in soft tissues. The small proportion of toxins excreted into water mostly constituted the free forms of the toxins. Both species shared the same route even though they contained very different proportions of free toxins in their soft tissues. No substantial changes in this general pattern were observed during the experiment. The esters of fatty acids with 16 carbon atoms were the most abundant in both soft tissues and faeces, but they were not the same in mussels and cockles. Most of the variability in ester proportions can be attributed to the species more than to their differential excretion (water or faeces) suggesting that there are not large differences in the depuration of the different esters.

## 1. Introduction

Harmful algal blooms (HABs) are natural phenomena that occur when toxic or noxious microalgae develop in the environment. Bivalves filter microalgae and accumulate the toxins they contain, thus becoming unsuitable for human consumption. More than 200 different taxa of microalgae are responsible for a wide range of HAB types worldwide [1]. In this context, and taking into account the exploitation of marine resources and rapid growth of aquaculture, food safety has become pivotal. Aquaculture surpassed capture fisheries for the first time in 2022, representing 51% of the world total [2].

Some dinoflagellates of the genera *Dinophysis* and *Prorocentrum* produce biotoxins that are involved in diarrhetic shellfish poisoning (DSP) [3,4,5,6,7,8,9,10,11], one of the most important foodborne diseases worldwide, together with paralytic (PSP) and amnesic (ASP) shellfish poisoning [12]. Human intoxication, with severe gastrointestinal syndrome, may happen after the consumption of contaminated shellfish with DSP toxins [3]. DSP has been recognised as a significant threat to public health worldwide, especially in Western Europe, where it seems to have significantly higher incidence in comparison with other world locations [5,13,14,15], perhaps due, at least in part, to the intense monitoring programmes which are active in this area [16]. In fact, in contrast to the global scenario, EU aquaculture is currently stagnated, in part by a decrease in the contribution of mussel production over the last two decades, predominantly associated with harmful algal blooms, and other factors such as microbial contamination and extreme weather events [17,18,19].

Originally extracted from the marine sponges *Halichondria okadai* and *H. melanodocia*, OA is an multiring polyether [20] that strongly inhibits serine/threonine protein phosphatases. These phosphatases are the primary targets for OA in mammalian tissues and the mechanism through which it triggers intestinal inflammation and diarrhoea in humans [21,22]. Additionally, OA interferes with various cellular functions, including cell growth and division, and can induce apoptosis. [23]. Structurally, OA and its analogues have a terminal carboxylic function and a hydroxyl group at C-7. The carboxylic group can esterify diols or other compounds. These groups of derivatives are named DTX4 to DTX7, and are usually found in producer cells [5,24,25,26,27,28,29]. The C-7 hydroxyl group is frequently esterified with fatty acids by bivalves and other organisms which ingest toxins to form a complex mixture of 7-O-acyl ester derivatives, generically known as DTX3 [30,31,32,33]. Due to their lipophilic nature, DSP toxins accumulate in soft tissues (mostly in the digestive gland [34]) of different bivalves of commercial interest, such as mussels, oysters, scallops, and clams (reviewed in [35]). The ratio of esterified to free toxins varies among species, typically being significantly higher in most shellfish, except for mussels and a small number of other species. [36,37,38,39,40,41,42,43,44,45]

The presence of okadaic acid and its analogues is relatively frequent in Europe [46]. In Galicia (NW Iberian Peninsula), the European area with the largest bivalve production, DSP episodes frequently produce bans on the harvesting and marketing of cultured mussels and (to a lesser extent) of other bivalves, thereby leading to important economic losses [36] and resulting in special interest in finding methods to mitigate the effects of these toxins. Some efforts have focused on the study of depuration acceleration by changing environmental conditions without achieving significant success (reviewed in [47]). The knowledge of depuration mechanisms therefore seems to be a key step in accelerating this process and reducing accumulation in bivalves. These mechanisms, however, have not been studied, and only the possible involvement of drug resistance proteins (MDR1 and MRP2) has been suggested in view of the overexpression of the genes that encode for these proteins in okadaic acid-exposed mussels [48]. Before studying the involved mechanisms, it is important to identify the depuration routes. Only a few studies have dealt with this subject, obtaining contradictory results, focusing on different routes, and using different methodologies, toxins, and species [33,49,50,51,52].

In this work, we studied the excretion of toxins into water and through faeces by two commercially important bivalve species (mussels and cockles), which can be expected to use different depuration routes because they esterify the toxins to a very different degree, with the aim of determining the main route of excretion of both free and esterified toxins, and the possible changes in the time course of the process.

## 2. Results

### 2.1. Total Toxin Depuration

**Figure 1:**
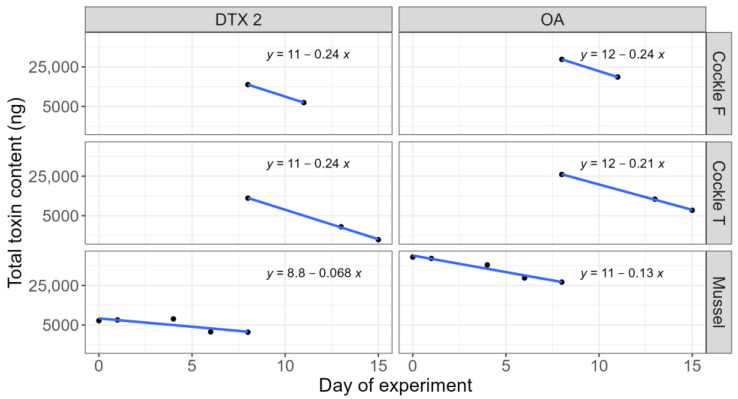
Variation in the total toxin content of the bivalves throughout the experiment (Cockle F = cockles obtained from O Freixo; Cockle T = cockles obtained from Testal). The lines and equations are the regression fitted to the logarithmically (natural logarithms) transformed data. The x coefficient is the estimated depuration rate in day^−1^.

During the experiment, the studied bivalves depurated a fraction of their toxins. The rate at which they lost the toxins depended on the toxins and the species. In cockles, the depuration rate was relatively fast, and very similar for OA and DTX2 (Figure 1). Even when the rate was estimated by only two points, the number of individuals at each sampling point made it a fairly reliable estimation of the exponential part of the depuration. In mussels, depuration was much slower, especially for DTX2, which was lost at approximately half of the velocity of OA.

### 2.2. Routes of Excretion

In general, considering the two species jointly, during the experiment, the total OA excreted with faeces was, on average, 20,882 ng/day, whereas only 423 ng/day was found in water (2% of the OA excreted via faeces). Free OA in faeces represented only a small proportion of the total OA excreted by that route (1.5%). The proportion found in the bivalves did not match that found in the major excretion route, with the free form of OA constituting 29% of the total. On average, free OA was excreted through faeces (311 ng/day) at a rate similar to that excreted into water (308 ng/day).

DTX2, like OA, was preferentially excreted in faeces. The average amount of this toxin excreted per day through faeces (4425 ng/day), which was much higher than that excreted into water (58 ng/day). The proportion of DTX2 excreted in the water (1.3%) was lower than that estimated for OA. In concordance with OA, bivalves contained a higher percentage of free DTX2 (31%) than their excreted products. In water, DTX2 was mostly present in its free form (80%).

**Figure 2:**
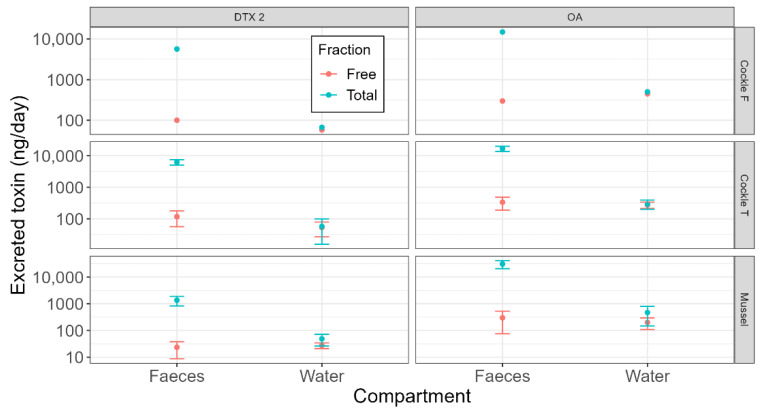
Total and free toxins excreted through faeces and water by the two species (mussels *Mytilus galloprovincialis* and cockles *Cerastoderma edule*) and the two cockle populations (Cockle F = cockles obtained from O Freixo; Cockle T = cockles obtained from Testal). The error bars represent the standard deviation.

**Figure 3:**
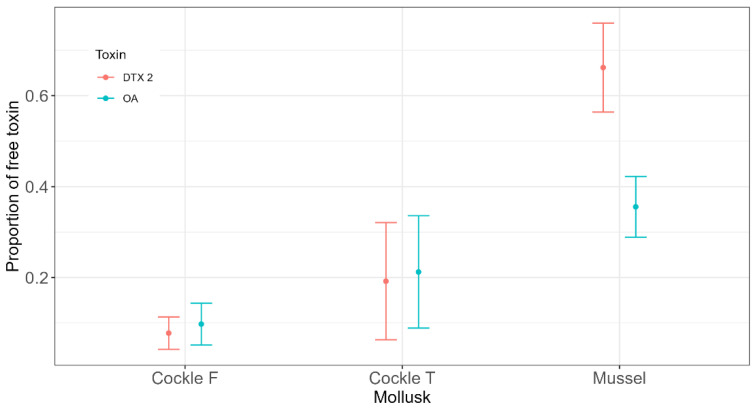
Proportion of free toxins in the soft tissues of mussels (*Mytilus galloprovincialis*) and the two cockle populations (Cockle F = cockles obtained from O Freixo; Cockle T = cockles obtained from Testal). The error bars represent the standard deviation.

The general pattern observed was very similar between the two bivalve species and between the two cockle populations (Figure 2). Most toxins were excreted through faeces in esterified form, and only a small percentage were excreted through water. Most of the toxins were, therefore, excreted as esters in both species, even when the average proportion of free forms in mussels was 0.36 for OA and 0.66 for DTX2, while in cockles, they were 0.15 for OA and 0.13 for DTX2, much lower than in mussels (Figure 3).

**Figure 4:**
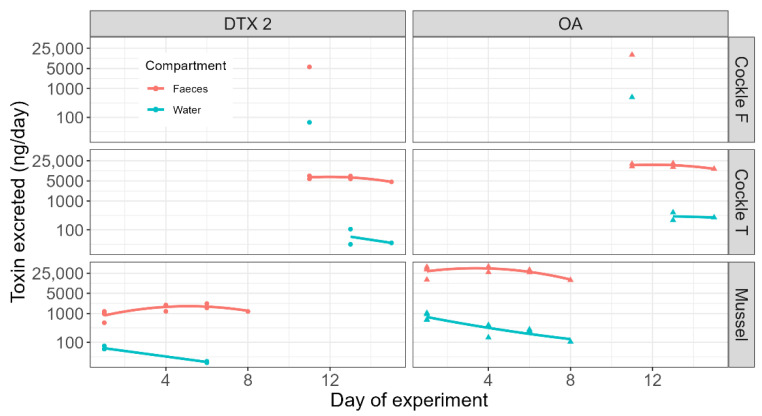
Total toxin excreted into water and faeces throughout the time course of the depuration of mussels and cockles (the collection and depuration of the species started at different dates) (Cockle F = cockles obtained from O Freixo; Cockle T = cockles obtained from Testal).

In general, a slight decreasing trend in the amount of toxins excreted/day over time was detected for both species and toxins (Figure 4). However, in faeces, a slight parabolic trend seems to be superimposed on the linear decrease, because excretion at intermediate times seems to be higher than expected. This parabolic trend was not observed in water. However, in faeces, a slight parabolic trend seems to be superimposed on the linear decrease because excretion at intermediate times seems to be higher than expected. This parabolic trend could not be detected in water.

**Figure 5:**
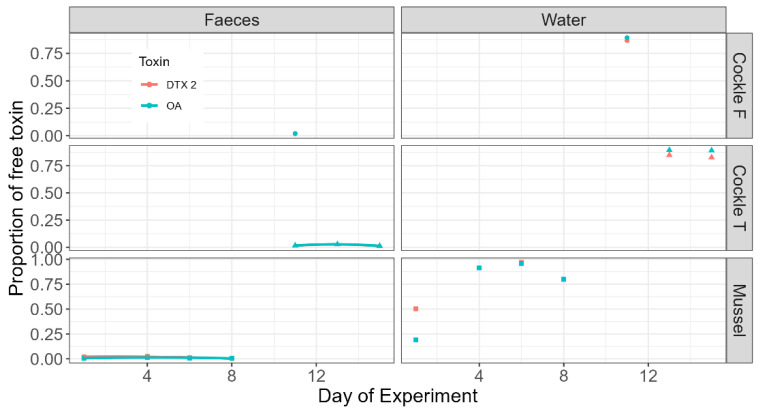
Proportion of free toxins in mussels and the two cockle populations in the experiment (Cockle F = cockles obtained from O Freixo; Cockle T = cockles obtained from Testal).

The proportion of free toxins in faeces during the experiment did not change substantially and was always very low (Figure 5). However, the proportion in the water was always high, with the exception of the first sampling day of mussels, when it was near or below 50%.

### 2.3. Excretion Products

**Figure 6:**
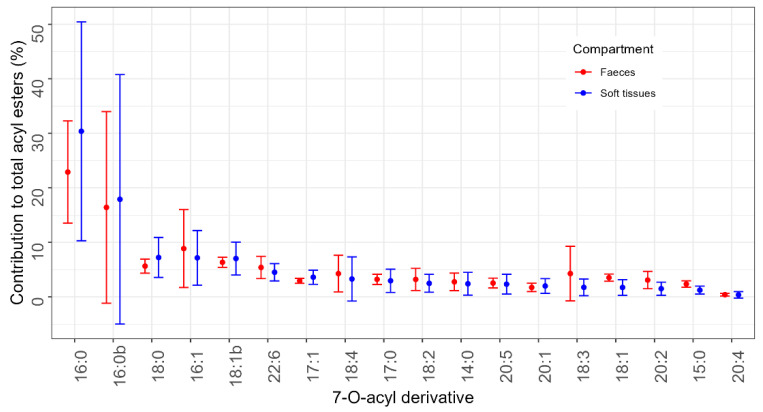
Contribution of the detected 7-O-acyl esters (the numbers indicate the carbon atoms and the degree of unsaturation of the esterifying fatty acid) to the total amount of these compounds in soft tissues and faeces. The contribution was computed assuming equal sensitivity of the method for all studied compounds. The error bars represent the standard deviation.

In general, fatty acids with 16 carbon atoms (two isomers of C16:0 and C16:1) seem to be the main esterifying compounds of OA and DTX2 (esters of these two toxins cannot be distinguished) in both soft tissues and faeces (Figure 6).

C16 fatty acid esters (palmitic acid and an isomer) were the main OA esters detected in both the soft tissues and faeces of the studied bivalves. There were differences in ester proportions between soft tissues and faeces, and between bivalve species. In general, the contribution of C16 fatty acid esters was lower in faeces than in soft tissues.

**Figure 7:**
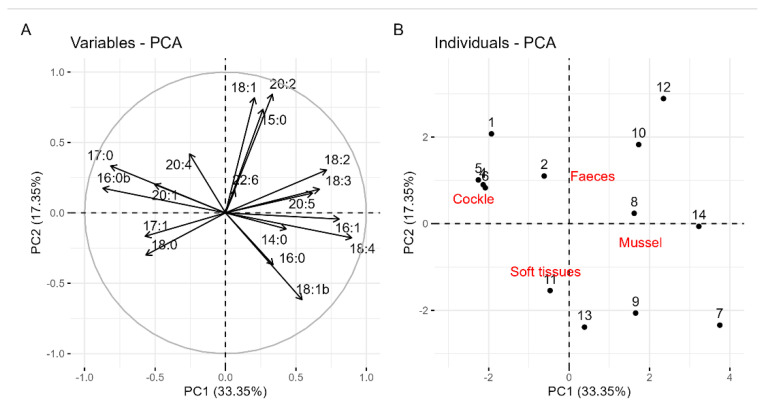
Principal component analysis of 7-O-acyl esters in soft tissues and faeces. The loadings of the esters on the first two principal components (with the percentage of variance explained in brackets) (the numbers of the arrows indicate the carbon atoms and the degree of unsaturation of the esterifying fatty acid) (**A**), and the scores of the observations, including the positions of the levels of the two qualitative auxiliary variables included (molluscs and routes) (**B**).

A principal component analysis of the proportions of esters in soft tissues and faeces showed that most of the observed variation (33.35% of the variance) was associated with species. The first principal component (PC1) is especially defined by the high negative loadings of esters C17:0 and C16:0b, and high positive loadings of C16:1 and C18:4. A smaller percentage of the variance was explained by PC2 (17.35%), a component related to the routes, which is mostly defined by high positive loadings of C18:1, C20:2, and C15:0 and moderate negative loadings of C18:1b and C16:0 (Figure 7).

**Figure 8:**
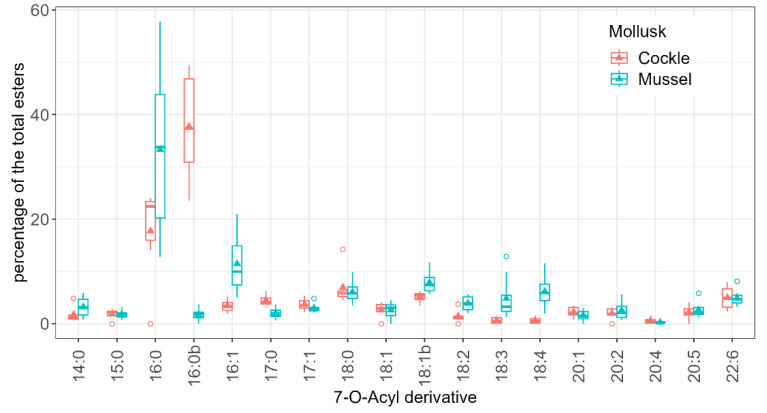
Contribution of the analysed 7-O-acyl esters of OA (the numbers indicate the carbon atoms and the degree of unsaturation of the esterifying fatty acid) to the total amount of these compounds in faeces and soft tissues (average) for the two species studied. The upper, middle, and lower lines in the boxes represent the 75, 50, and 25% percentiles. The extremes of the vertical lines are the largest and smallest values once outliers are excluded, which are represented as dots. The triangles represent the means.

The most striking difference between species is the presence of a palmitic acid ester isomer in cockles, which replaces the palmitic ester found in mussels (Figure 8).

**Figure 9:**
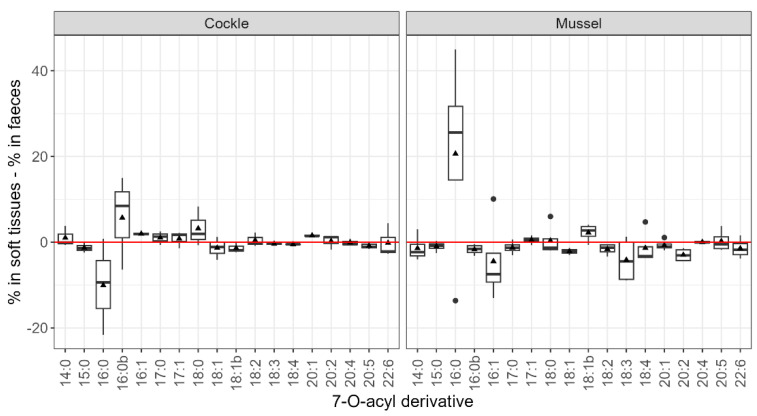
Differences between soft tissues and faeces in the percentages of the total esters in each route represented by the studied acyl esters (the numbers indicate the carbon atoms and the degree of unsaturation of the esterifying fatty acid). The upper, middle, and lower lines in the boxes are the 75, 50, and 25% percentiles. The extremes of the vertical lines are the largest and smallest values once outliers are excluded, which are represented as dots. The triangles are the means and dots represent outliers.

The proportion of the total that each ester represents in soft tissues and faeces was significantly different for the two species (ANOVA, *p* = 0.025), and also between esters in mussels, but only the differences between C16:0 and C16:0b, C18:1, and C20:2 were statistically significant (Tukey, *p* < 0.05). Apart from these significant differences, several other compounds were proportionally more abundant in soft tissues than in faeces (Figure 9). The most important esters, due their concentration, are probably C16:0 and C16:0b in cockles and C16:0 and C16:1 in mussels.

## 3. Discussion

In our study, both bivalve species mostly excreted OA and DTX2 (>98%) through faeces and after acylation with fatty acids. This result supports our previous findings, which pointed to this route as the main route of elimination of these toxins in the mussel *M. galloprovincialis* [33,49,53], and those of Marcaillou et al. in *M. edulis* [51]. A study by Nielsen et al. [50], however, through the intoxication of the mussel *M. edulis* with *Dinophysis acuta* in laboratory conditions, suggested that the elimination of free OA in its dissolved form was the main excretion route in sight of the mass balance of the toxin during depuration. Unfortunately, excretion through faeces was not measured in that experiment. Another experiment carried out by Matsushima et al. in a similar way, but studying the accumulation and excretion of DTX1 from scallops fed with *Dinophysis fortii* [52], obtained similar results. In that case, additionally, very low excretion of DTX1 was found and mostly in free form. We do not have a clear explanation for these discrepancies. Metabolic differences between the species do not seem to be responsible, because even the usual percentage of esterified toxins is similar (it seems higher in the case of DTX1 in scallops [54]). It is possible that, in the experiments which fed *Dinophysis* to the bivalves, most of the toxin excreted during the first days of depuration came from emptying the digestive system of broken dinoflagellate cells, more than from the already absorbed toxin. This is supported by the high depuration (nearly one-third of the accumulated toxins) during the first day of the Nielsen et al. experiment, instead of approximately 13%, which was estimated for the entire experiment. However, this could not explain the toxin balance found in the experiment. The findings from our study aligned with those of Nielsen et al. and Matsushima et al., indicating that the majority of toxins detected in the dissolved state were present in their free form rather than as esterified compounds. In our case, some esters were found, but their presence could be due to the amount of time they were maintained in the culture tanks and the processes used to recover faeces, which can favour the leaching of these compounds into the water.

There were no substantial differences in the routes of excretion between the two species studied, even when they accumulated esterified toxins at very different proportions, nor between the two toxins studied, which were not esterified to the same extent by mussels. This suggests that excretion of the esterified toxins is the main depuration route and that toxins are eliminated from the bivalves through this route, even if considerable amounts of free toxins are present in the organism. Lindegarth et al. [44] found a similar depuration rate of total OA in mussels and oysters, but a substantially lower apparent depuration rate of OA esters in mussels, in which the percentage of esterified OA was much lower. This difference in apparent depuration can be explained by the elimination of the esters, but with their replacement by means of the transformation of free OA in mussels and pure depuration of the esters, without being compensated by transformation (because most OA was already transformed) in oysters. The slow depuration rate of DTX2 relative to OA in mussels (found in this experiment, but also in other cases [36,55]) seems to be due to its lower proportion of esters, which are the forms that are mainly excreted.

Principal Component Analysis (PCA) revealed that species differences accounted for most of the variation in individual ester proportions. This variation can be primarily attributed to the differences between mussels and cockles. Specifically, cockles exhibited higher percentages of a C16:0 isomer (C16:0b) and C17:0, whereas mussels showed greater proportions of C16:1, C18:2, C18:3, and C18:4. Differences in acyl-ester profiles between the two species have already been found by Vale [56] in Portugal, showing that cockles accumulate more branched esters and esters with odd carbon numbers than mussels. Only a small percentage of the variability was linked to the route, suggesting that, in general, the differences in acyl-esters between faeces and soft tissues are not large, and consequently, that there is likely no clear preferential elimination of any particular acyl ester. Torgersen et al. [57] did not find any important difference in the OA- and DTX1-ester profiles during the depuration of the mussel *M. edulis* and the oyster *Ostrea edule*. However, in our study, some compounds were found to be relatively more abundant in soft tissues than in faeces, which suggests that these esters are produced at a higher rate by molluscs or that they are excreted more slowly. Among these are C16:0 in mussels and 16:0b in cockles. In the opposite situation, there are C15:0, C18:1, and C20:2 in both species; C18:2, C18:3, C18:4, and C16:1 in mussels; and C16:0 in cockles. The opposite behaviour of C16:0 and C16:0b in cockles suggests a possible transformation between them. It seems more likely that the higher contribution of C16:0 in mussels was due to a high rate of esterification of the toxins with palmitic acid, which is the most abundant fatty acid in the mussel *M. galloprovincialis* [58,59,60], and was found to be dominant during the early phases of intoxication [61]. The same seems to be true for C16:0b (the C16:0 isomer) in cockles, which was found to be abundant [56]. Further studies are required to identify the causes of the observed changes in the proportions.

It seems, therefore, that to accelerate depuration, activating the chain-free toxin-esterified toxin in faeces seems to be a promising approach. There are two key steps that seem to be different for the two species studied. The first one, esterification, seems to be more important in regulating depuration in mussels, especially DTX2, because the proportion of esterified toxins rarely approaches 100% and probably limits the amount of toxin that could be excreted. The second step, the excretion of esters into faeces, seems to be the process limiting depuration in cockles because nearly all toxins are quickly esterified. Accelerating these two steps would probably increase the depuration velocity not only in the two studied species, but also in others that have similar esterification characteristics.

## 4. Materials and Methods

### 4.1. Biological Material and Experimental Design

Mussels (*M. galloprovincialis*) (shell length = 7.44 ± 0.55 cm) were obtained from a culture raft in Ría de Vigo, Galicia, NW Spain, during an active DSP episode. Cockles (*C. edule*) were obtained from two different locations in Ría de Muros: Testal (shell length = 2.86 ± 0.20 cm) and O Freixo (shell length = 2.81 ± 0.22 cm), at a later date but during the same active episode. Mussels were randomly distributed into five groups of ten individuals/group. One group was used to determine the initial toxin concentration, and the other four groups were subjected to the experimental conditions. Because of the limited number of cockles available, their smaller size, and their lower faeces production, they were distributed in a different way than the mussels. The initial toxin levels were determined by randomly selecting 10 individuals from each location for analysis. The experimental conditions were then applied to the remaining specimens. For O Freixo, the entire group of 62 individuals was collectively treated. In contrast, the Testal sample was randomly divided into two groups, each containing approximately 60 individuals, before undergoing the experimental procedures. Each group was enclosed in a net with a 2 cm mesh and suspended in a 5 L tank filled with 4 L of filtered seawater aerated by means of a diffusor placed inside a test tube to allow oxygenation but to limit turbulence and faeces breakdown. A continuous supply of phytoplankton culture, mostly composed of *Tetraselmis*, T-iso, and *Chaetoceros*, was distributed to the tanks using an Ismatec peristaltic pump. As mussels and cockles were not obtained on the same date and because of the different numbers of individuals and their expected faecal production, the experiment was carried out in two phases. During the first experiment, the mussels distributed in the four tanks were monitored. During the second phase (which started 8 days after the first one), cockles from the two sampling locations distributed in three tanks (one for O Freixo and two for Testal) were studied. In each phase, on the defined sampling dates, the bivalves were removed from the tank and, after allowing the faeces to settle, a 100 mL water sample was taken and centrifuged at 4000× *g*, and the supernatant was frozen until extraction and analysis. Subsequently, most of the water in the tank was carefully discarded to keep the faeces at the smallest possible volume. Then, the suspended faeces were centrifuged at 4000× *g* (using four 250 or 500 mL bottles), the supernatants were frozen (only to check the magnitude of the losses during the process), and the pellets were combined and centrifuged again in a 50 mL tube. The obtained pellet was then extracted with MeOH (see extraction and hydrolysis section) and maintained at −20 °C until further processing and analysis. Soft tissues of the bivalves from one of the tanks were obtained, weighed, and extracted. The extracts were maintained at less than −20 °C until analysis. The remaining tanks were filled again with 4 L of filtered seawater, and the bivalves were suspended until the next sampling date. The mussel experiment started on day 0, and the mussels were sampled on days 1, 4, 6, and 8. The cockle experiments started on day 8. The cockles from O Freixo were sampled on day 11, and those from Testal on days 11 (only water and faeces), 13, and 15.

### 4.2. Reagents

LC-MS-grade MeOH from VWR Chemicals (Llinars del Vallés, Spain) was used for extraction. For alkaline hydrolysis, 35% NaOH and HCl, both for analysis, from Merck (Madrid, Spain) were used. Ultrapure water obtained from a Milli-Q gradient (Merck Millipore, Madrid, Spain), as well as Supelco LC-MS-grade acetonitrile and NH4OH, both obtained from Merck (Madrid, Spain), were used for the chromatographic phases. For quantification, certified reference materials for OA, DTX1, and DTX2 were obtained from Laboratorio CIFGA (Lugo, Spain).

### 4.3. Extraction and Hydrolysis

The soft tissues of the bivalves were homogenised with a blade homogenizer, and a 2 g aliquot was obtained. The aliquot was extracted twice with MeOH at a proportion of 5:1 *w*:*v* using an Ultraturrax (IKA, Germany). The obtained suspensions were centrifuged at >4000× *g* and the supernatants were combined. The faeces obtained from the pellets were extracted by adding 4 mL of MeOH, centrifuging at >4000× *g*, recovering the supernatant, repeating the process, and combining the two supernatants. The final volume was made up to 10 mL using MeOH. Toxins were extracted from the water using two methods. The first, for free toxins (raw samples), was an online solid-phase extraction, injecting 50 µL of the obtained water directly into the chromatographic system in a SecurityGuard AJO 8367 precolumn for Gemini NX C18 (Phenomenex, Alcobendas, Spain), following the method of Regueiro et al. [62]. The second method, for total toxins (after alkaline hydrolysis), was used because it was not possible to perform alkaline hydrolysis of the esters of the toxins in water. Aliquots of the water samples collected on the same day were combined, and 20 mL was extracted through a Strata-X SPE column (Phenomenex), washed with Milli-Q water, and eluted with 2 mL of acetonitrile. The eluate obtained was concentrated using a SpeedVac (Thermo Scientific, Alcobendas, Spain) and then reconstituted in 300 µL of MeOH. A 100 µL aliquot of this solution underwent standard alkaline hydrolysis before analysis. The recoveries of both methods exceeded 80%.

### 4.4. LC-MS/MS Analysis

The samples were analysed with a binary UHPLC system, Sciex (Alcobendas, Spain) Exion AD, coupled to a Sciex QTRAP 6500+ hybrid triple quadrupole and ion trap mass spectrometer through an IonDrive Turbo V interface operated in electrospray mode. Chromatographic separation was performed in a Phenomenex Gemini NX C18 50 × 2.1 mm, 3 µm, using the method of Regueiro et al. [62] but slightly modifying it in two different ways, for free toxins and for acyl-esters (Table 1). The methods used an on-line SPE system made of a SecurityGuard AJO 8367 precolumn placed in a 6-way, 2-position valve that selects the input and output lines of the SecurityGuard and an external Jasco (Madrid, Spain) PU-2080 pump that delivered a mixture of chromatographic phases A and B in a proportion of 90:10.

In both methods, mobile phase A was water and phase B was acetonitrile/water 90:10, both with 6.7 mM ammonium hydroxide; the flow rate was set to 0.4 mL/min. The injection volume used was 1 µL for soft tissues and faeces, and 50 µL for water.

The mass spectrometer was operated in negative and positive polarisation modes for the free toxins and for esters, respectively. The interface was operated with a temperature of 600 °C, nominal Gas 1 and Gas 2 at 75, spray voltages of 5000 v and −4500 v, and curtain gas 30. The analysis of free forms (OA and DTX2) was carried out in MRM mode using the transitions 803.5 > 255.1 and 803.5 > 563.3 with CEs of 62 and 60, respectively. The 7-O-acyl esters were also analysed in MRM mode using the transitions from the precursors in Table 2 to the fragment with *m/z* 809.5, in all cases with a CE of 62.

Quantification was carried out by external calibration using two sets of calibrating solutions: one for faeces and soft tissues, and another one, diluted in a proportion of 1:50 with filtered and OA- and DTX2-free seawater, for water samples. For OA and DTX2 in tissues and faeces, and water, the LOQs were 0.14 ng/mL and 18 pg/mL, respectively. Data were acquired using Sciex Analyst 1.7.3, and processed using Sciex OS 3. As it was not possible to directly determine the total toxins in water, pooled water samples from each sampling day were subjected to SPE extraction, as described in Section 4.3. The concentrations of total toxins in the water were estimated by multiplying the concentrations of free toxins obtained by direct analysis by the ratio of the total to free toxins obtained from these samples. Additionally, the four water samples from the first day of the mussel experiment were individually analysed.

### 4.5. Statistical Analysis

All statistical tests were performed with R (version 4.3.0) [63]. Plots were made using the ggplot2 (version 3.4.2) R package [64], and principal component analysis was conducted with the packages FactoMineR (version 2.8) [65] and factoextra (version 1.0.7) [66].

## Figures and Tables

**Table 1:** Gradients used for the determination of free toxins and esters. Valve indicates the position of the 6-way 2-position valve. Position 1 allows us to inject the sample into the precolumn while, through a parallel flow, the analytical column equilibrates. Position 2 diverts the flow of the analytical pump to the precolumn and then to the analytical column.

Free Toxins			Esters		
Time (min)	%B	Valve	Time (min)	%B	Valve
0	22	1	0	10	1
2.5	22	2	2.5		2
4	54		4	10	
4.75	95		5.5	25	
5.25		1	6.5	70	
6.5	95		8.5	80	
8	22		9	95	
			10		1
			11.5	95	
			12	10	
			13	10	

**Table 2:** Transitions used to determine the main 7-O-acyl esters (esters of OA and DTX2 cannot be distinguished). Sodium adducts were used as precursors. The compound names indicate the carbon number and double bonds of the acyl chains.

Compound	Precursor *m/z*	Compound	Precursor *m/z*
Na-14:0	1037.501	Na-18:2	1089.5
Na-15:0	1051.5	Na-18:3	1087.501
Na-16:0	1065.5	Na-18:4	1085.501
Na-16:1	1063.5	Na-20:1	1119.5
Na-17:0	1079.5	Na-20:2	1117.5
Na-17:1	1077.5	Na-20:4	1113.5
Na-18:0	1093.5	Na-20:5	1111.5
Na-18:1	1091.5	Na-22:6	1137.5

## Data Availability

Due to technical/time limitations, the raw data supporting the conclusions of this article will be made available by the authors on request.
